# Five New Iridoids from Roots of *Salvia digitaloides*

**DOI:** 10.3390/molecules191015521

**Published:** 2014-09-29

**Authors:** Shwu-Jen Wu, Yu-Yi Chan

**Affiliations:** 1 Department of Medical Laboratory Science and Biotechnology, Chung Hwa University of Medical Technology, Tainan 71703, Taiwan; 2 Department of Biotechnology, Southern Taiwan University of Science and Technology, Tainan 71005, Taiwan; E-Mail: yuyichan@mail.stust.edu.tw

**Keywords:** *Salvia digitaloides*, Labiatae, iridoid glucosides

## Abstract

Five new iridoids, salvialosides A–E (compounds **1**–**5**), together with fifty known compounds were isolated from the roots of *Salvia digitaloides*. The structures of the new compounds were completely elucidated using a combination of 2D NMR techniques (COSY, NOESY, HMQC and HMBC) and HR-ESI-MS analyses. The known compounds were identified by comparison of their spectroscopic and physical data with those reported in the literature.

## 1. Introduction

**Figure 1:**
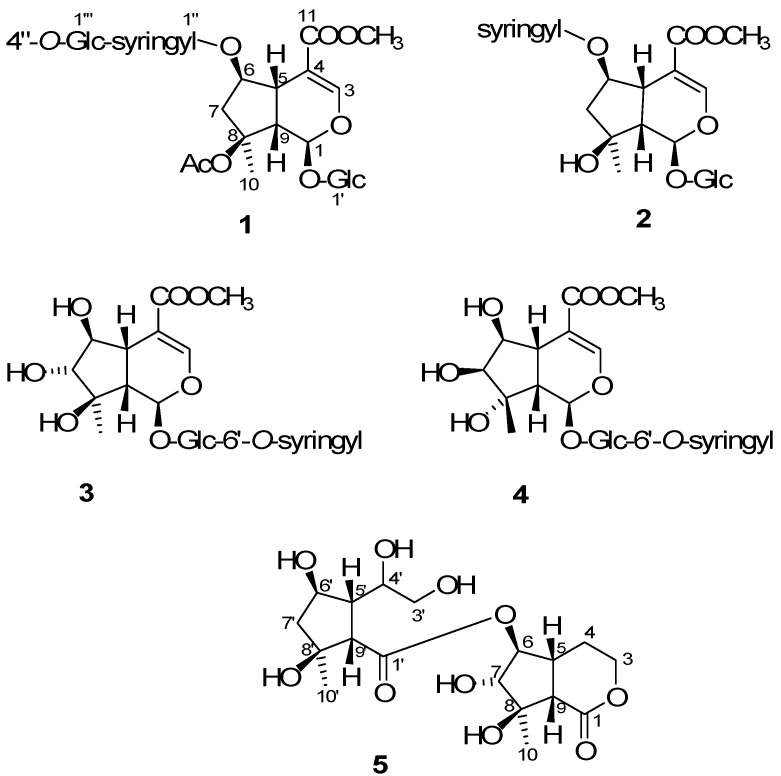
Structure of the new compounds **1**–**5**.

*Salvia* is the largest genus in the economically and medicinally important family Labiatae [1], due to its many interesting biological and pharmacological activities, including antitumor [2], antiallergic [3], antioxidant [4], antimicrobial [5], and antiplatelet aggregation effects [6]. *Salvia digitaloides* which is an herbaceous perennial shrub native to the Chinese provinces of Guizhou, Sichuan, and Yunnan, has been used in traditional Yunnan medicine. The local Tibetans soak the roots of this plant in alcohol to manufacture a special traditional health drink, claimed to make them physically strong [7]. Although the isolation of some iridoid glycosides from *Salvia digitaloides* had been published in the previous literature [8,9], only diterpenes were isolated from *S. digitaloides* in our previous study. In order to explore the constituents of the roots of *S. digitaloides*, we have continued to study the constituents of this plant. In this paper, we report the isolation and structural determination of five new iridoids, salvialosides A–E (compounds **1**–**5**, Figure 1), from the roots of *S. digitaloides*, together with fifty known compounds.

## 2. Results and Discussion

### 2.1. Purification and Characterization

The dried roots of *S. digitaloides* were extracted with methanol under reflux to give a deep brown syrup. The crude extract was subjected to purification by a combination of conventional techniques to afford five new iridoids (compounds **1**–**5**). In addition, fifty known compounds were isolated and identified as chysophanol (**6**) [10], glutinol (**7**) [11], physcion (**8**) [10], β-sitosterone (**9**) [11], β-sitosterol (**10**) [11], emodin (**11**) [12], vanillin (**12**) [13], 5α,8α-epidoxy-24-ethyl-cholesta-6-22-dien-3-β-ol (**13**) [14], ergosta-4,6-dien-3-one (**14**) [15], asperphenamate (**15**) [16], oleanolic acid (**16**) [17], 7-oxo-β-sitosterol (**17**) [18], salviatalin A (**18**) [19], salvitrijudin A (**19**) [19], syringic acid (**20**) [13], baiynoside (**21**) [9], phlomisosides II (**22**) [8], colchiside A (**23**) [20], stachlic acid (**24**) [21], salviatalin A-19-*O*-β-glucoside (**25**) [22], glycerol (**26**) [23], 2,3-dihydro-2-(4-hydroxy-3-methoxyphenyl)-3-hydroxymethyl-7-methoxybenzofuran-5-carboxylic acid (**27**) [24], 7,8-dimethylisoalloxazine (**28**) [25], ferulic acid (**29**) [13], 2-phenylethyl-O-β-glucoside (**30**) [26], 6-*O*-syringyl-8-*O*-acetylshanzhiside methyl ester (**31**) [9], salviadigitoside A (**32**) [22], 4-hydroxybenzoic acid (**33**) [13], methyl-3-(3-hydroxyphenyl)-propanoate (**34**) [27], vanillic acid (**35**) [13], malic acid (**36**) [28], 8-*O*-acetylshanzhiside methyl ester (**37**) [9], verbascoside (**38**) [29], caffeic acid (**39**) [30], leucosceptoside A (**40**) [31], syringoylglycerol glucoside (**41**) [32], 1-methyl-β-carboline-3-carboxylic acid (**42**) [33], forsythoside B (**43**) [29], phlorigidoside C (**44**) [29], shanzhiside methyl ester (**45**) [9], phloyoside II (**46**) [29], 7,8-dehydropentstemoside (**47**) [34], cistanoside D (**48**) [35], tryptophan (**49**) [36], 1-ribitol-2,3-diketo-1,2,3,4-tetrahydro-6,7-dimethylquinoxaline (**50**) [37], salviadiginine A (**51**) [22], 7-epiphlomiol (**52**) [34], 4-hydroxybenzaldehyde (**53**) [13], hydroxytyrosol (**54**) [38], and jioglutolide (**55**) [39] by comparison of their physical and spectral data with those reported in the corresponding literature.

### 2.2. Structural Elucidation of Compounds **1**–**5**

**Figure 2:**
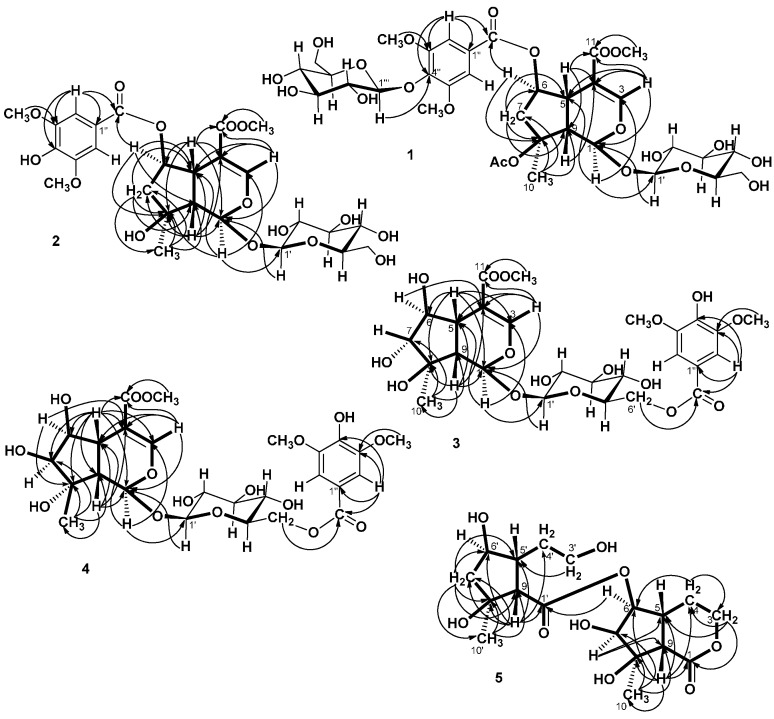
HMBC correlations of **1**−**5**.

**Figure 3:**
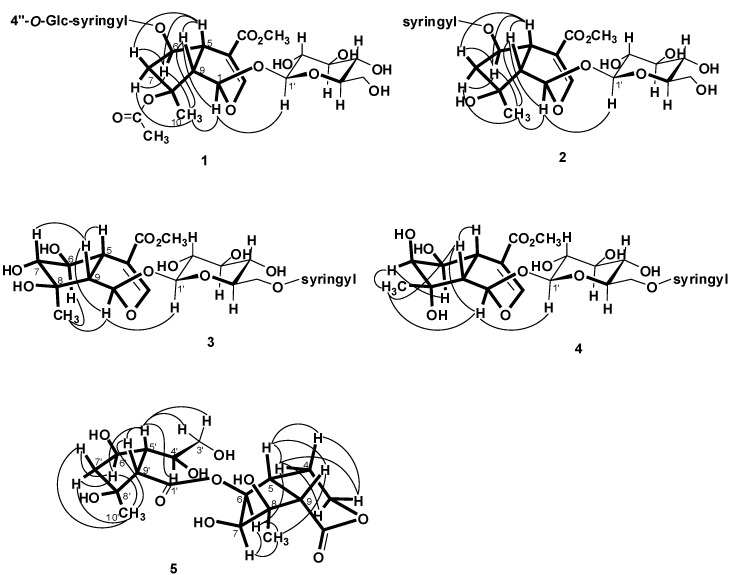
NOE correlations of **1**−**5**.

Salvialoside A (**1**) was isolated as an optically active colorless amorphous powder with 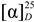 = −36.0 (*c* 0.35, MeOH). The HR-ESIMS showed a molecular ion at *m/z* 813.2424, corresponding to the molecular formula C_34_H_46_O_21_Na. The UV spectrum revealed a very broad absorption band at 263 nm and the IR spectrum exhibited absorption maxima at 3367, 1705 and 1635 cm^−1^, indicating the presence of hydroxyl, α,β-unsaturated carbonyl and phenyl groups. The ^1^H- and ^13^C-NMR spectra showed great similarity with those of iridoid glucoside, 6-*O*-syringyl-8-*O*-acetylshanzhiside methyl ester (**31**). In the NMR spectrum (Table 1), signals for iridoid nucleus presented an enol ether unit [δ_H_ 7.57 (H-3); δ_C_ 154.5 (C-3), 108.6 (C-4)] with a carbomethoxy group [δ_H_ 3.67 (OCH_3)_; δ_C_ 168.4 (C-11), 51.9 (OCH_3_)], an oxygenated methine [δ_H_ 5.51 (H-6); δ_C_ 79.9 (C-6)], a dioxygenated methine [δ_H_ 5.90 (H-1); δ_C_ 95.3 (C-1)], two aliphatic methine [δ_H_ 3.09 (H-9), 3.48 (H-5); δ_C_ 50.4 (C-9), 40.1 (C-5)], a methylene group [δ_H_ 2.27, 2.51 (H-7); δ_C_ 45.0 (C-9)], and a methyl group [δ_H_ 1.63 (H-10); δ_C_ 21.8 (C-10)]. Moreover, the iridoid structure was completely verified by the following HMBC correlations (Figure 2): H-1 with C-3/C-5, H-3 with C1/C-4/C-5/C-11, H-5 with C-4/C-9/ C-11, H-6 with C-4/ C-8, H-7 with C-5/C-6/C-8/C-9, and H-10 with C-7/C-8/C-9. The *cis*-fused cyclopentanodihydropyran ring system in iridoid was confirmed by the strong NOE correlation (Figure 3) between H-5 and H-9. In addition, a,β-glucopyranosyl moiety was characterized by the NMR signals at δ_H_ 4.71 (d, *J* = 8.0 Hz, H-1') and δ_C_ 62.5 (C-6'), 71.4 (C-4'), 74.7 (C-2'), 77.9 (C-3'), 78.5 (C-5'), and 100.2 (C-1'). The HMBC correlations (Figure 2) of H-1 with anomeric C-1' and anomeric H-1' with C-1, and the NOE correlation (Figure 3) between iridoid H-1 and glucose H-1' indicated a structure of iridoid 1-glucoside. A set of NMR signals at δ_H_ 7.38 (2H, s, H-2" and -6") and 3.93 (6H, s, 3"- and 5"-OCH_3_) as well as δ_C_ 57.3 (3"- and 5"-OCH_3_), 108.7 (C-2" and -6"), 127.4 (C-1"), 140.6 (C-4"), 154.3 (C-3" and -5"), and 166.8 (C=O) assigned to a syringic acid which made an ester linkage with 6-OH inferred at first from the deshielded H-6 (δ 5.51) and confirmed by the HMBC correlation of H-6 with syringyl C=O. The stereochemistry of H-5, H-9, and 1-*O*-glucosyl was supposed to adopt universally β-configuration. By careful examination of the NOESY spectrum, the β-face orientations of the 6-*O*-syringyl and 8-*O*-acetyl were proved by the presence of weak NOE correlation between the 8-CH_3_ (H-10) and H-6/H-1/H-7(α). In addition, the unexpected weak NOE correlations between H-9(β) and H-10(α), H-5(β) and H-6(α) were occasionally observed and it was noted that Jensen *et al.* [40] and Ersoz *et al.* [41] have claimed that in some conformations they were close in space and should indeed give rise to such NOEs. The remaining ^1^H- and ^13^C-NMR signals showed the other set of glucose at δ_H_ 5.13 (d, *J* = 7.8 Hz, H-1'") and δ_C_ 63.0 (C-6'"), 71.6 (C-4'"), 75.7 (C-2'"), 77.9 (C-3'"), 78.5 (C-5'"), and 104.3 (C-1'"). Again, the large coupling constant, 7.8 Hz, between H-1'" and H-2'" was in agreement with a β-glucose as the sugar unit.

The HMBC correlation between H-1'" and C-4" indicated the glucosylation should be at C-4" of syringate. Hence, a 6-*O*-(4"-*O*-glucopyranosyl)syringyl-8-*O*-acetylshanzhiside methyl ester was deduced for **1** based on the above analysis and given the trivial name salvialoside A.

**Table 1:** ^1^H- and ^13^C-NMR spectroscopic data for compounds **1**–**4** in CD_3_OD.

Position	1				2				3				4			
	δ_H_ (*J* in Hz)		δ_C_		δ_H_ (*J* in Hz)		δ_C_		δ_H_ (*J* in Hz)		δ_C_		δ_H_ (*J* in Hz)		δ_C_	
1	5.90 d (3.6)		95.3		5.55 d (4.8)		93.9		5.56 d (1.4)		92.4		5.48 d (2.2)		95.2	
3	7.57 d (1.4)		154.5		7.47 d (1.0)		152.1		7.38 d (1.2)		150.9		7.41 d (1.2)		153.2	
4			108.6				110.7				111.1				111.9	
5	3.48 ddd (8.8, 2.9, 1.4)		40.1		3.52 ddd (9.0, 3.6, 1.0)		37.1		2.70 ddd (11.2, 4.8, 1.2)		34.3		2.82 ddd (10.9, 5.2, 1.2)		38.3	
6	5.51 dd (5.6, 2.9)		79.9		5.52 ddd (7.4, 4.2, 3.6)		78.4		3.48 dd (8.0, 4.8)		80.5		4.06 dd (5.2, 4.4)		79.5	
7	α: 2.27 dd (15.4, 5.6) β: 2.51 d (15.4)		45.0		β: 1.93 dd (14.0, 4.2) α: 2.34 dd (14.0, 7.4)		46.4		3.73 d (8.0)		84.4		3.60 d (4.4)		81.2	
8			89.8				79.6				76.7				80.8	
9	3.09 dd (8.8, 3.6)		50.4		2.54 dd (9.0, 4.8)		50.6		2.53 dd (11.2, 1.4)		45.7		2.54 dd (10.9, 2.2)		47.6	
10	1.63 s		21.8		1.37 s		21.9		1.03 s		16.5		1.27 s		23.1	
11			168.4				167.7				168.7				170.6	
OCH_3_	3.67 s		51.9		3.56 s		50.4		3.72 s		50.8		3.73 s		52.0	
8-OAc	1.92 s		22.3, 172.6													
1'	4.71 d (8.0)		100.2		4.71 d (8.0)		98.9		4.66 d (8.0)		98.3		4.69 d (8.0)		100.2	
2'	3.22 dd (9.2, 8.0)		74.7		3.20 dd (9.0, 8.0)		73.4		3.19 dd (9.2, 8.0)		73.4		3.21 dd (9.2, 8.0)		74.6	
3'	3.30–3.49 m		77.9		3.37 t (9.0)		74.6		3.41 m		76.6		3.41 m		77.8	
4'	3.30–3.49 m		71.4 ^a^		3.22 t (9.0)		70.6		3.41 m		70.4		3.41 m		71.8	
5'	3.30–3.49 m		78.5		3.35 m		76.6		3.62 m		74.6		3.68 m		75.8	
6'	3.66 dd (12.0, 5.6) ^c^ 3.79 dd (12.0, 2.3) ^c^		62.5 ^b^		3.64 dd (11.8, 6.3) 3.92 dd (11.8, 2.4)		63.8		4.39 dd (12.0, 5.4) 4.66 dd (12.0, 2.0)		63.8		4.44 dd (12.0, 6.1) 4.66 dd (12.0, 2.1)		65.0	
1"			127.4				120.7				120.0				121.2	
2", 6"	7.38 s		108.7		7.39 s		108.7		7.33 s		107.1		7.35 s		108.3	
3", 5"			154.3				147.8				147.8				148.9	
4"			140.6				140.6				140.6				142.1	
1"-C=O			166.8				166.8				166.8				167.9	
3",5"-OCH_3_	3.93 s	57.3		3.89 s		55.7		3.89 s		55.7		3.88 s		56.9		
1'"	5.13 d (7.8)	104.3														
2'"	3.52 dd (9.2, 7.8)	75.7														
3'"	3.30–3.49 m	77.9														
4'"	3.30–3.49 m	71.6 ^a^^'^														
5'"	3.30–3.49 m	78.5														
6'"	3.69 dd (12.2, 5.6) ^c'^ 3.94 dd (12.0, 2.2) ^c'^	63.0 ^b'^														

^a^ and ^a'^, ^b^ and ^b'^, ^c^ and ^c'^: Assignments may be interchangeable. m: Overlapping or irresolvable peak. ^1^H- and ^13^C-NMR data (δ) were measured in CD_3_OD at 400 and 100 MHz.

Salvialosides B (**2**) was obtained as optically active colorless amorphous powder. The HR-ESIMS at *m/z* 609.1791, suggested the molecular formula C_26_H_34_O_15_Na. All the IR, UV, and ^1^H- and ^13^C-NMR spectral data (Table 1) were similar to those of 6-*O*-syringyl-8-*O*-acetylshanzhiside methyl ester (**31**), an iridoid 1-glucoside with a syringyl group. The esterification site was determined to be at C-6 by the downfield-shifted H-6 (δ 5.52) and the HMBC correlation (Figure 2) of H-6 (δ 5.52) with syringyl C=O (δ 166.8). The difference between **2** and **31** was the substituent on C-8. In **2**, a hydroxyl substituent, instead of an acetoxy group, on C-8 was observed by the upfield-shifted C-8 (δ from 88.3 in **31** to 79.6 in **2**). Despite the presence of NOE interactions (Figure 3) between H-9 and H-10/H-1, and between H-5 and H-6, the β-orientations of the syringyl, hydroxyl, and glucosyl groups were confirmed by the the presence of weak NOE correlation between H-10 and H-6/H-1/H-7(α). Consequently, the iridoid glucoside **2** was identified as salvialosides B. It is a new compound from a natural source, although it has been obtained previously by the mild hydrolysis of 6-*O*-syringyl-8-*O*-acetylshanzhiside methyl ester (**31**) with NaOH in methanol [9].

Salvialosides C (**3**) had the molecular formula C_26_H_34_O_16_Na determined from HR-ESIMS at peak *m/z* 625.1740. In the ^1^H-NMR spectrum (Table 1), compared with **2**, the upfield-shifted H-6 (δ from 5.52 to 3.48) and the downfield-shifted H-7 (δ from 2.34 and 1.93 to 3.73) reflected each C-6 and C-7 connected a hydroxyl group. In the ^13^C-NMR spectrum (Table 1), except 11 signals for iridoid monoterpene and six signals for glucose by comparing those with known iridoid 1-glucosides, the remaining six signals at δ 55.7, 107.1 (2 × C), 120.0, 140.6, 147.8 (2 × C), and 166.8 combined with ^1^H-NMR signals at δ 3.89 (6H) and 7.33 (2H) were almost identical to the syringly signals. The downfield-shifted H-6' (δ 4.39 and 4.66) suggested that the syringic acid with C-6' hydroxyl of glucose formed an ester. The HMBC correlation of glucosyl H-6' with syringyl C=O (δ 166.8) further confirmed this connection. The stereochemistry of the aglycone of **3** was determined by coupling and NOE data (Figure 3). H-3 showed a long-range coupling (1.2 Hz) with H-5 which, in turn, showed ^3^*J* couplings of 11.2 and 4.8 Hz with H-9 and H-5, respectively. Therefore a large coupling constant of 8.0 Hz between H-6 and H-7 indicated the two protons should be oriented in a pseudo-axial direction. In addition, the significant NOE correlations between H-9 and H-1/H-5/H-7 and between H-10 and H-6 together with the absence of NOE correlations between H-5 and H-6 as well as between H-9 and H-10 suggested that the three hydroxyls as β-6-OH, α-7-OH, and β-8-OH and they all located toward pseudo-equatorial direction. Consequently, the structure of compound **3** was assigned as salvialoside C.

Salvialosides D (**4**), of molecular formula C_26_H_34_O_16_Na according to the molecular ion peak at *m/z* 625.1724 in HR-ESIMS, was thus indicated to be an isomer of **3**. The ^1^H-NMR and ^13^C-NMR signals (Table 1) were close related to those of **3** with an iridoid monoterpene, a glucose, and a syringic acid. The syringic acid formed an ester functionality with glucose by the HMBC correlation between H-6' (δ 4.44 and 4.66) of glucose and C=O (δ 167.9) of the syringyl group. The difference between **3** and **4** was the stereochemistry of C-7 and C-8. The small coupling constant of 4.4 Hz between H-6 and H-7, together with the absence of NOE correlation between H-7 and H-5/H-9 indicated was H-7 orientated in a pseudo-equatorial direction (α-face), whereas the existence of strong NOE correlations between H-10 and H-7/H-9 and the absence of NOE correlation between H-10 and H-6 suggested a,β-H-10. The absence of NOE correlation between H-5 and H-6 confirmed the α-face of H-6. The β-6-OH, β-7-OH, and α-8-OH thus established. Therefore, compound **4** was identified as 7,8-epi-salvialoside C, and named salvialoside D.

Salvialoside E (**5**) was yielded as colorless powder with the molecular formula C_18_H_28_O_10_Na by the HR-ESIMS signal at *m/z* 427.1582. The IR bands at 3383, 1747, 1708 cm^−1^ revealed the hydroxyl and carbonyl functions. The presence of an iridoid moiety and a six-membered-ring-opened iridoid moiety was deduced according to 1D and 2D NMR spectral analysis. From the ^1^H-NMR (Table 2) and COSY spectra, the iridoid moiety consisted of an oxygenated ethyl unit at δ 4.25 and 4.50 (H-3) and 1.82 and 2.05 (H-4), four mutually-coupled methines at δ 3.03 (H-9), 2.43 (H-5), 4.28 (H-6), and 3.56 (H-7) along with a methyl at δ 1.50 (H-10). Compared with iridoids **1**–**4**, the C-1 hemiacetal was oxidized to an ester (δ_C_ 174.8) and the double bond between C-3 and C-4 was reduced to a single bond (δ_C_ 67.5 and 26.4, respectively). A δ-lactone ring existed by the HMBC correlation (Figure 2) between H-3 and C-1. This six-membered ring was verified *cis*-fused to a five-membered ring by the strong NOE correlation (Figure 3) between H-5 and H-9. A long-range W type coupling (1.2 Hz) presented between H-5 and H-3 (eq) together with the NOEs correlations between H-5 and two H-4 suggested that H-5 located at the equatorial direction of the δ-lactone ring. The presence of NOE correlations between H-10 and H-6/H-7, H-7 and H-6/H-10 and the absence of NOE correlation between H-7 and H-5/H-9 suggested that 6-OR, 7-OH, and 8-OH all orientated toward the β-face. On the other hand, the six-membered-ring-opened iridoid part was a cyclopentane ring with a carboxylic group (δ_C_ 178.3, C-1') on C-9', dihydroxyl ethyl group (δ_H_ 3.64 and 3.78 and δ_C_ 64.7 for H-3', δ_H_ 4.32 and δ_C_ 85.1 for H-4') on C-5', except a hydroxyl group on C-6' as well as a hydroxyl and a methyl groups on C-8'.

**Table 2:** ^1^H- and ^13^C-NMR spectroscopic data for compound **5** in CD_3_OD.

Position	δ_H_ (*J* in Hz)	δ_C_
1		174.8
3	β (eq): 4.25 dtd (11.2, 3.6, 1.2) α (ax): 4.50 td (11.2, 2.3)	67.5
4	α (eq): 1.82 dtd (11.2, 3.6, 2.3) β (ax): 2.05 tdd (11.2, 6.5, 3.6)	26.4
5	2.43 m	40.8
6	4.28 dd (8.3, 3.8)	77.8
7	3.56 d (3.8)	81.2
8		83.0
9	3.03 d (11.5)	51.4
10	1.50 s	22.5
1'		178.3
3'	3.64 dd (12.2, 3.8) 3.78 dd (12.2, 2.9)	64.7
4'	4.32 td (3.8, 2.9)	85.1
5'	3.00 ddd (9.6, 3.8, 3.0)	52.5
6'	4.17 dt (5.0, 3.0)	78.8
7'	1.88 dd (13.8, 5.0) 1.96 ddd (13.8, 3.0, 1.2)	48.4
8'		82.1
9'	3.17 dd (9.6, 1.2)	58.2
10'	1.43 s	24.5

^1^H- and ^13^C-NMR data (δ) were measured in CD_3_OD at 400 and 100 MHz.

The HMBC correlations of H-9' with C-1', H-9' with C-4', and H-3' with C-5' proved their attachment. The NOE correlations between H-10' and H-6' verified the two protons positioned toward α-face. Finally, the two parts were linked together by an ester functionality as H-6 showed the HMBC correlation with C-1'. Consequently, the bis-iridoid structure of **5** was established as salvialoside E.

## 3. Experimental Section

### 3.1. General Information

All the chemicals were purchased from Merck KGaA (Darmstadt, Germany) unless specifically indicated. Melting points of purified compounds were determined by a Yanagimoto MP-S3 melting point measuring apparatus without correction. UV spectra were obtained on a Hitachi UV-3210 spectrophotometer (Hitachi, Tokyo, Japan). IR spectra were recorded on a Shimadzu FTIR spectrometer Prestige-21 (Shimadzu, Tokyo, Japan). Optical rotations were measured using a Jasco DIP-370 Polarimeter (Jasco, Tokyo, Japan). Electrospray ionization (ESI) and HRESI mass spectra were recorded on a Bruker APEX II mass spectrometer (Bruker, Rheinstetten, Germany). The NMR spectra, including ^1^H-NMR, ^13^C-NMR, COSY, NOESY, HMBC, and HSQC experiments, were recorded on Bruker Avance 400 and AV-500 NMR spectrometers (Bruker) with TMS as the internal reference, and chemical shifts are expressed in δ (ppm). Silica gel (Merck, 70–230, 230–400 mesh) was used for column chromatography and thin layer chromatography (TLC) was conducted on pre-coated Kiesel gel 60 F_254_ plates (Merck), and the spots were visualized by UV.

### 3.2. Plant Materials

The roots of *S. digitaloides* were collected from in Li Jiang, Yunnan Province, People’s Republic of China, in October 2004 by S. Zhang, Institute of Materia Medica, Chinese Academy of Medicinal Sciences, Beijing, China, and identified by Kuoh, C. S. Department of Life Sciences, National Cheng Kung University, Tainan, Taiwan. Permission was obtained to export the plant material from China to Taiwan. A voucher specimen (TSWu-20041015) was deposited at the Herbarium of National Cheng Kung University.

### 3.3. Extraction and Isolation

The dried roots of *S. digitaloides* (3.0 kg) were pulverized into powder and extracted six times with methanol (10 L) for 8 h under reflux. The methanol soluble extract was concentrated under reduced pressure to give a dark brown syrup (220 g). The methanol-soluble extract was suspended in water and then extracted with chloroform and *n*-BuOH successively to afford the chloroform layer (71 g), *n*-BuOH fraction (68 g). The CHCl_3_ layer was chromatographed on silica gel, eluted with a mixture of chloroform and methanol (19:1, 9:1, 7:1, 5:1, 3:1, 1:1) to give eight subfractions (Fr. 1–8). Fr. 3 was subjected to silica gel column chromatography (CC) eluted by solvent mixture of *n*-hexane and ethyl acetate (49:1) to yield chysophanol (**6**, 3.4 mg), glutinol (**7**, 4.1 mg), physcion (**8**, 4.7 mg), β-sitosterone (**9**, 11.3 mg) and β-sitosterol (**10**, 120.6 mg). Fr. 4 was purified by silica gel CC with a mixing eluent of *n*-hexane and ethyl acetate (49:1) to yield emodin (**11**, 2.3 mg), vanillin (**12**, 2.3 mg), 5α,8α-epidoxy-24-ethyl-cholesta-6-22-dien-3-β-ol (**13**, 7.3 mg), ergosta-4,6-dien-3-one (**14**, 4.2 mg) and asperphenamate (**15**, 3.1 mg). Fr. 5 was subjected to silica gel CC eluted by solvent mixture of chloroform and methanol (49:1) to produce oleanolic acid (**16**, 6.3 mg), 7-oxo-β-sitosterol (**17**, 2.8 mg), salviatalin A (**18**, 5.2 mg) and salvitrijudin A (**19**, 2.1 mg). Fr. 6 was purified by silica gel CC with a mixing eluent of chloroform and methanol (19:1) to yield syringic acid (**20**, 6.4 mg), and baiynoside (**21**, 23.3 mg).

The *n*-BuOH fraction was chromatographed over reversed-phase Diaion HP-20 gel using water and methanol gradients (water: methanol = 1:0, 9:1, 5:1, 3:1, 2:1, 1:1, 1:3, 1:9, 0:1) and afforded eight fractions according to the TLC monitoring. Fraction 2 was subjected to silica gel column chromatography with a gradient of ethyl acetate and methanol (5:1), to produce baiynoside (**21**, 1.1 g) and phlomisosides Π (**22**, 16 mg). Fraction 3 was chromatographed on silica gel eluted with a mixture of chloroform and methanol (3:1) to yield colchiside A (**23**, 3.2 mg), stachlic acid (**24**, 1.5 mg), salviatalin A-19-*O*-β-glucoside (**25**, 4.7 mg), glycerol (**26**, 1.9 mg), and 2,3-dihydro-2-(4-hydroxy-3-methoxyphenyl)-3-hydroxymethyl-7-methoxybenzofuran-5-carboxylic acid (**27**, 1.1 mg). Fraction 4 was purified by column chromatography over silica gel with a mixture of chloroform and methanol (3:1) to yield salvialoside B (**2**, 17.3 mg), 7,8-dimethylisoalloxazine (**28**, 2.3 mg), ferulic acid (**29**, 2.1 mg), 2-phenylethyl-*O*-β-glucoside (**30**, 1.4 mg), and 6-*O*-syringyl-8-*O*-acetylshanzhiside methyl ester (**31**, 830.0 mg). Fraction 5 was chromatographed on silica gel eluted with a mixture of chloroform and methanol (5:1) to produce salvialoside C (**3**, 1.2 mg), salvialoside D (**4**, 31.6 mg), and salviadigitoside A (**32**, 2.1 mg). Fraction 6 was subjected to SiO_2_ CC eluted by a solvent mixture of chloroform and methanol (5:1) to yield salvialoside A (**1**, 3.5 mg), syringic acid (**20**, 3.2 mg), 4-hydroxybenzoic acid (**33**, 8.1 mg), methyl-3-(3-hydroxyphenyl)propanoate (**34**, 2.7 mg), vanillic acid (**35**, 1.5 mg), malic acid (**36**, 3.1 mg), 8-*O*-acetylshanzhiside methyl ester (**37**, 4.2 mg), verbascoside (**38**, 87 mg), caffeic acid (**39**, 2.3 mg), leucosceptoside A (**40**, 3.2 mg), syringoylglycerol glucoside (**41**, 1.2 mg), 1-methyl-β-carboline-3-carboxylic acid (**42**, 9.9 mg), and forsythoside B (**43**, 3.3 g). Fraction 7 was isolated by SiO_2_ CC eluted with mixture of chloroform and methanol (5:1) to yield phlorigidoside C (**44**, 1.8 mg), shanzhiside methyl ester (**45**, 3.7 mg), phloyoside II (**46**, 11.0 mg) and 7,8-dehydropentstemoside (**47**, 4.8 mg), cistanoside D (**48**, 10.1 mg), tryptophan (**49**, 53.6 mg), 1-ribitol-2,3-diketo-1,2,3,4-tetrahydro-6,7-dimethylquinoxaline (**50**, 4.3 mg), salviadiginine A (**51**, 5.1 mg), and 7-epiphlomiol (**52**, 8.2 mg). Fraction 8 was subjected to SiO_2_ CC eluted by solvent mixture of chloroform and methanol (7:1) to afford salvialoside E (**5**, 3.9 mg), 4-hydroxybenzaldehyde (**53**, 1.0 mg), hydroxytyrosol (**54**, 5.2 mg), and jioglutolide (**55**, 42.2 mg), respectively.

#### 3.3.1. Salvialoside A (**1**)

Colorless amorphous powder; 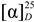 = −36.0 (*c* 0.35, MeOH); UV (MeOH), λ_max_ (log ε) 216 (3.95), 263 (3.61) nm; IR(KBr) ν_max_: 3367, 1705, 1635 cm^−1^; ^1^H- and ^13^C-NMR see Table 1 ; ESIMS *m/z* (rel. int.): 813 [M+Na]^+^; HRESIMS *m/z*: 813.2424 [M+Na]^+^ (calcd. for C_34_H_46_O_21_Na, 813.2429).

#### 3.3.2. Salvialoside B (**2**)

Colorless amorphous powder; 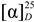 = −103.4 (*c* 0.99, MeOH); UV (MeOH), λ_max_ (log ε) 220 (4.41), 277 (4.01 ) nm; IR(KBr) ν_max_: 3367, 1701 cm^−1^; ^1^H- and ^13^C-NMR see Table 1; ESIMS *m/z* (rel. int.): 609 [M+Na]^+^; HRESIMS *m/z*: 609.1791 [M+Na]^+^ (calcd. for C_26_H_34_O_15_Na, 609.1795).

#### 3.3.3. Salvialoside C (**3**)

Colorless syrup; 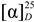 = −80.0 (*c* 0.24, MeOH); UV (MeOH), λ_max_ (log ε) 220 (4.60), 277 (4.16) nm; IR(KBr) ν_max_: 3379, 1693 cm^−1^; ^1^H- and ^13^C-NMR see Table 1; ESIMS *m/z* (rel. int.): 625 [M+Na]^＋^; HRESIMS *m/z*: 625.1740 [M+Na]^＋^ (calcd. for C_26_H_34_O_16_Na, 625.1742).

#### 3.3.4. Salvialoside D (**4**)

Colorless amorphous powder; 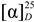 = −14.5 (*c* 0.42, MeOH); UV (MeOH), λ_max_ (log ε) 220 (4.51), 276 (4.07) nm; IR(KBr) ν_max_: 3329, 1697 cm^−1^; ^1^H- and ^13^C-NMR see Table 1; ESIMS *m/z* (rel. int.): 625 [M+Na]^＋^; HRESIMS *m/z*: 625.1742 [M+Na]^＋^ (calcd. for C_26_H_34_O_16_Na, 625.1744).

#### 3.3.5. Salvialoside E (**5**)

Colorless amorphous powder; 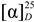 = −0.80 (*c* 0.48, MeOH); UV (MeOH), λ_max_ (log ε) 217 (3.41) nm; IR(KBr) ν_max_: 3383,1747, 1708 cm^−1^; ^1^H- and ^13^C-NMR see Table 2; ESIMS *m/z* (rel. int.): 427 [M+Na]^＋^; HRESIMS *m/z*: 427.1580 [M+Na]^＋^ (calcd for C_18_H_28_O_10_Na, 427.1582).

## 4. Conclusions

In our investigation, fifty five compounds were isolated from the roots of *Salvia digitaloides*, including twelve iridoid glycosides, seven diterpenoids, seven triterpenoids, four caffeic acid sugar esters, and other compounds. Among, them the salvialosides A–E (compounds **1**–**5**) were new compounds isolated for the first time as natural products.
